# A Review of Pediatric Heel Pain

**DOI:** 10.7759/cureus.34228

**Published:** 2023-01-26

**Authors:** Ezan A Kothari, Anthony M Padgett, Sean M Young, Jessyca Ray, Ashish Shah, Michael J Conklin

**Affiliations:** 1 Orthopaedic Surgery, University of Alabama at Birmingham School of Medicine, Birmingham, USA

**Keywords:** sever's apophysitis, tumors, infections, inflammation, pediatrics, heel pain

## Abstract

The objective of this review article is to provide orthopaedic surgeons and general practitioners a reference and guidance for the evaluation and workup of heel pain in pediatric patients. The authors performed a comprehensive literature search to review the etiologies and management of heel pain in patients <18 years of age. Relevant studies in Medline/PubMed and EMBASE were searched from inception to March 3, 2022 using medical subject headings and text words without limitations on language or study type. The initial search utilized the following Boolean operators: (children) AND (heel pain); (pediatric) AND (heel pain). Heel pain in the pediatric population is usually a benign condition. Sever’s apophysitis is the most common etiology of heel pain in pediatric patients. Most causes of heel pain in the pediatric population do not require imaging or extensive workup. However, providers must maintain a high index of suspicion for symptoms that could indicate a more severe pathology.

## Introduction and background

Heel pain is a common musculoskeletal complaint likely to be encountered in a pediatric orthopedic practice. A study of 1000 pediatric appointments found that 8.2% of visits were due to heel pain [1]. Sever’s apophysitis, one of the most common causes of heel pain, has been estimated to comprise anywhere from 2% to 16% of all pediatric musculoskeletal complaints [2].

It is often challenging to determine the true etiology of heel pain and therefore a general understanding of the evaluation of the child with heel pain is critical for determining which cases need further evaluation and which do not. This review will cover the workup, diagnosis, and management of the most common and worrisome causes of heel pain in the pediatric population with particular emphasis on overuse conditions, congenital abnormalities, fractures, infections, benign lesions, and malignant lesions.

## Review

Clinical evaluation

The clinical evaluation of heel pain consists of four components: history, physical examination, imaging, and laboratory studies. 

History

Once the main complaint has been established, important questions should be asked. These include the location of pain, duration, antecedent trauma or puncture wound, aggravating and alleviating factors, relationship of pain to weight bearing, constitutional symptoms such as fever and weight loss, and previous treatment. 

Physical Examination 

The examination begins with the measurement of vital signs, including temperature. There are four major components of the examination: inspection, palpation, range of motion, and gait assessment. Inspection involves evaluation of the standing position of the foot as well as assessment for erythema, swelling, or deformity. The foot is palpated for calor, tenderness, or masses. Range of motion of the ankle (dorsiflexion/plantarflexion) and subtalar joint (inversion/eversion) is examined and compared to the opposite foot or to normal range of motion in bilateral cases. Gait is then evaluated. Toe walking or an antalgic gait where the patient shortens the stance phase on the involved side is common. The Silfverskiold test is used to evaluate for tightness of the gastrocnemius-soleus muscular complex. 

Imaging

**Figure 1:**
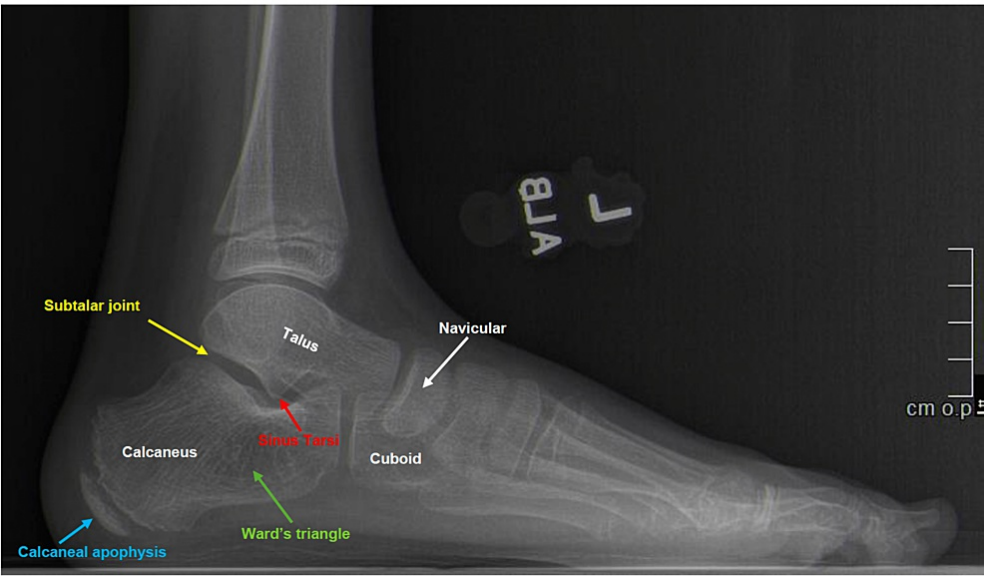
Lateral weightbearing radiograph of the foot. Major anatomical landmarks of the hindfoot are labeled including the calcaneus and its surrounding structures. The calcaneus articulates with the bone directly superior to it, the talus, via the subtalar (or talocalcaneal) joint. The sinus tarsi is a “canal” located at the lateral ankle that is formed between the talus and calcaneus. The calcaneal apophysis is part of the growing portion of the calcaneus that acts as a site of attachment for the Achilles tendon. This is one of the most common sites of heel pain in children. Ward’s triangle is a hypodense region within the calcaneus that is more susceptible to fracture and can be the site of calcaneal cyst formation.

Important anatomic structures appreciated on plane radiographs are demonstrated in Figure 1. Within the subtalar joint, the posterior facet of the calcaneus is responsible for bearing the majority of the weight of the talus. The talus also articulates with the calcaneus anteriorly via the smaller anterior calcaneal facet. 

Imaging begins with routine radiographs. This typically involves standing X-rays of the foot. An axial view of the calcaneus known as a Harris heel view can be helpful, particularly in cases of suspected tarsal coalition. In cases of bilateral heel pain in a child within the typical age group for Sever’s calcaneal apophysitis (eight to 15 years of age), radiographs are generally unnecessary. 

Advanced imaging can be considered for specific indications. In cases where there has been puncture with a non-radiopaque foreign body, ultrasound may confirm the presence or absence of a retained foreign body. Computed topography (CT) scan is useful to evaluate tarsal coalition. Magnetic resonance imaging (MRI) is the advanced imaging study of choice for infection, neoplasm, or stress fracture not visible on routine radiograph [3,4].

Laboratory Evaluation

Laboratory evaluation including complete blood count (CBC), erythrocyte sedimentation rate (ESR), and C-reactive protein (CRP) should be performed for patients with constitutional symptoms.

Specific entities

Calcaneal Apophysitis (Sever’s Apophysitis)

**Figure 2:**
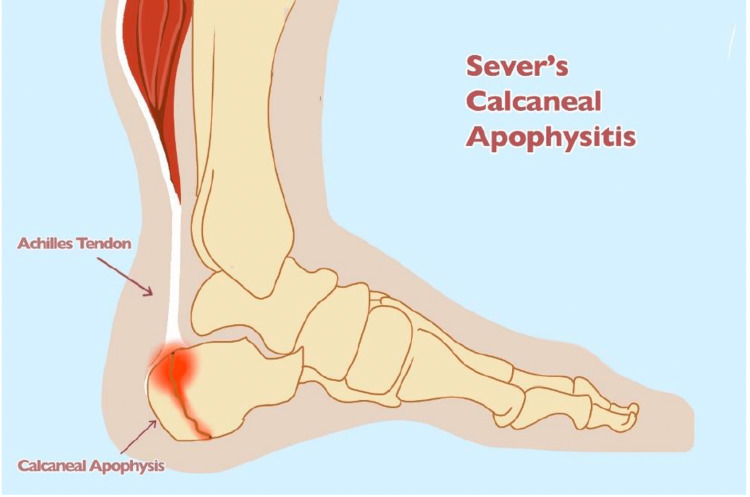
Sever’s apophysitis. Sever's calcaneal apophysitis is pain at the Achilles tendon insertion into the calcaneal apophysis.

Calcaneal apophysitis, or Sever’s apophysitis, was first described by Sever in 1912 as an inflammatory condition at the insertion of the Achilles tendon to the calcaneus (Figure 2) [5]. 

An apophysis is a growth center at a tendon insertion into bone (Figures 1, 2). Various apophyses can become painful in children at specific ages. Since the calcaneal growth plate does not usually close before 14 years of age, the typical age at which Sever’s apophysitis presents ranges from eight to 15 years old [6-9]. When pediatric patients present with this condition, they complain of insidious unilateral or bilateral heel pain that is usually associated with activity. There are no constitutional symptoms.

**Figure 3:**
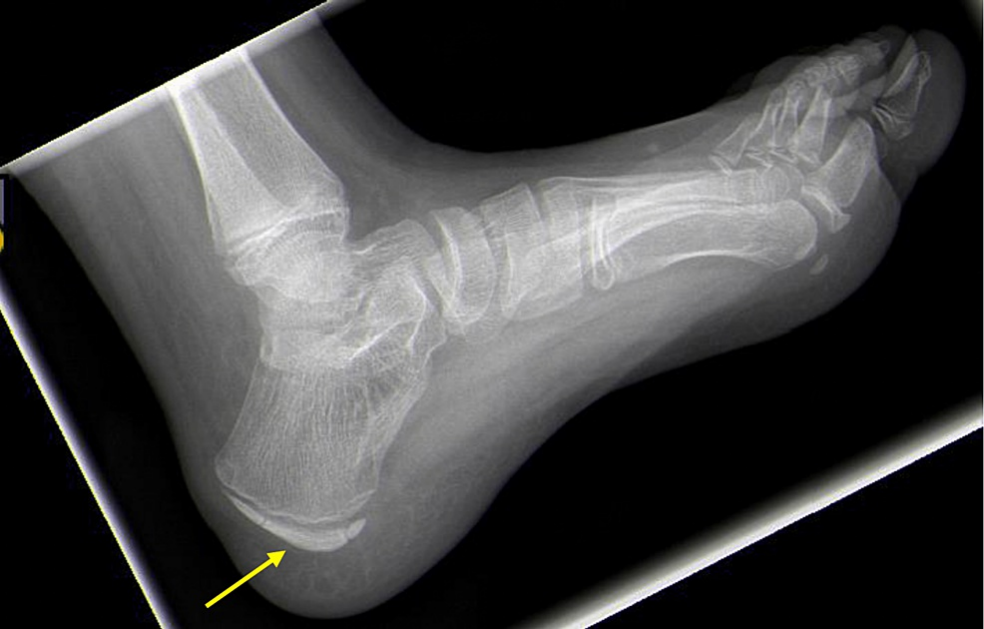
Lateral radiograph of the foot in a patient with Sever’s apophysitis. Note sclerosis and fragmentation of the calcaneal apophysis, which is not specific to Sever’s apophysitis and can be observed in asymptomatic patients.

On physical examination, there is tenderness to palpation directly over the apophysis at the posterior aspect of the heel. Children may walk on their toes or limp to avoid placing pressure on the heel. In addition, examination of the gastrocnemius-soleus complex via the Silfverskiold test may reveal tightness of this complex noted by decreased dorsiflexion of the ankle with the knee extended in comparison to the amount of dorsiflexion achieved with the knee flexed at 90 degrees. Radiographs are not indicated in patients complaining of bilateral heel pain when Sever’s apophysitis is suspected. However, in unilateral cases, radiographs should be obtained to rule out other causes of heel pain [10]. Typically, radiographs are normal, and clinical exam is sufficient for diagnosis (Figure 3). 

Treatment of calcaneal apophysitis is focused on symptomatic relief. Effective conservative treatment consists of a combination of Achilles stretching, heel cup shoe inserts, NSAIDs, and activity modification [9, 11]. Wiegerinck et al. demonstrated that conservative measures such as activity modification, physical therapy, and orthotic use resulted in significant improvement in pain; however, no treatment was demonstrated to be superior to the others [12]. Sever’s apophysitis can last up to 2 years, serving as a significant source of parental frustration and worry. Therefore, it is important to reassure parents that their child’s condition will resolve without sequelae and the utilization of conservative treatment should improve their child’s symptoms significantly.

Tarsal Coalition

Tarsal coalition is an autosomal dominant condition in which two or more tarsal bones are fused together by either fibrous tissue, cartilage, bone, or a combination [13-15]. It is speculated that tarsal coalitions result from incomplete differentiation and segmentation of the mesenchyme. The most common tarsal coalition subtypes include calcaneovnavicular and talocalcaneal coalitions, which constitute approximately 90% of all coalitions [16]. The true incidence is unknown as most patients are asymptomatic; however, some authors suggest that tarsal coalitions are present in about 1 to 15% of the general population [13,14,17].

Patients typically begin exhibiting symptoms associated with tarsal coalition between the ages of eight and 16 [14,15,17]. Patients will complain of vague pain around the anterior and lateral aspect of the ankle that is deep and aching in nature. This pain is exacerbated by activity and relieved by rest. Recurrent lateral ankle sprains may also be noted in the patient’s history [14,15].

On physical examination, there may be increased hindfoot valgus, but varus can also be seen. It is important to assess subtalar motion in these patients with particular care to ensure that the motion is indeed coming from the subtalar joint instead of the talonavicular or calcaneocuboid joints, which may become hypermobile in the presence of coalitions. Examination for subtalar motion is performed by locking the talus into the mortise with neutral ankle dorsiflexion while passively inverting and everting the heel [14,15]. The patient can also be asked to stand on tiptoes while the examiner observes the heel from behind. In a normal scenario, the heel should invert whereas in tarsal coalition, the heel will remain in valgus. Tenderness is present over the coalition as well as the dorsal aspect of talonavicular joint [15].

**Figure 4:**
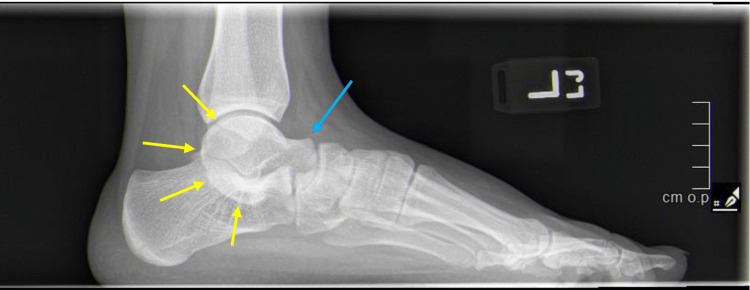
Lateral weightbearing radiograph of the left foot. Radiograph demonstrates signs of talo-calcaneal tarsal coalition including beaking of the talus (blue arrow) and “C-sign” (yellow arrows).

Plain radiographs are useful for diagnosing tarsal coalition. With calcaneonavicular coalitions, the anterior process of the calcaneus is elongated on the lateral view, the so-called “anteater sign”. The oblique view is confirmatory. In talocalcaneal coalition, the lateral radiograph may show a “C-sign”, which corresponds to bridging between the talar dome and sustentaculum tali; however, it must be noted that the “C-sign” is not pathognomonic (Figure 4) [13,15,17]. A Harris heel view can be confirmatory for talocalcaneal coalition. For either type of coalition, lateral radiographs may disclose “beaking” of the talus that corresponds to a traction spur on the dorsal head of the talus (Figure 4) [14, 15, 18]. First-line treatment for tarsal coalition is non-operative management. Surgical resection is indicated for persistently symptomatic coalitions. CT can be utilized to localize and determine the extent of coalitions and should be performed for pre-operative planning. MRI may be useful to evaluate for suspected fibrous or cartilaginous coalition that cannot be seen on routine radiographs or CT. 

Plantar Fasciitis

Plantar fasciitis is an overuse syndrome caused by repetitive stress and trauma to the plantar fascia that connects the calcaneal tuberosity to the proximal phalanges on the plantar aspect of the foot [19,20]. In the pediatric population, plantar fasciitis can be seen in any age group, particularly in children who participate in sports that involve high levels of running, jumping, and inclines [20,21]. Plantar fasciitis is usually seen in combination with Sever’s apophysitis in the young athlete. However, it may present in isolation with medial heel and arch pain in the adolescent patient whose physes have already closed [20]. In a study of 1000 pediatric visits, heel pain was found to be responsible for 8.2% of visits. Of those, 40% were attributed to plantar fasciitis, or an incidence of 3.3% [1].

**Figure 5:**
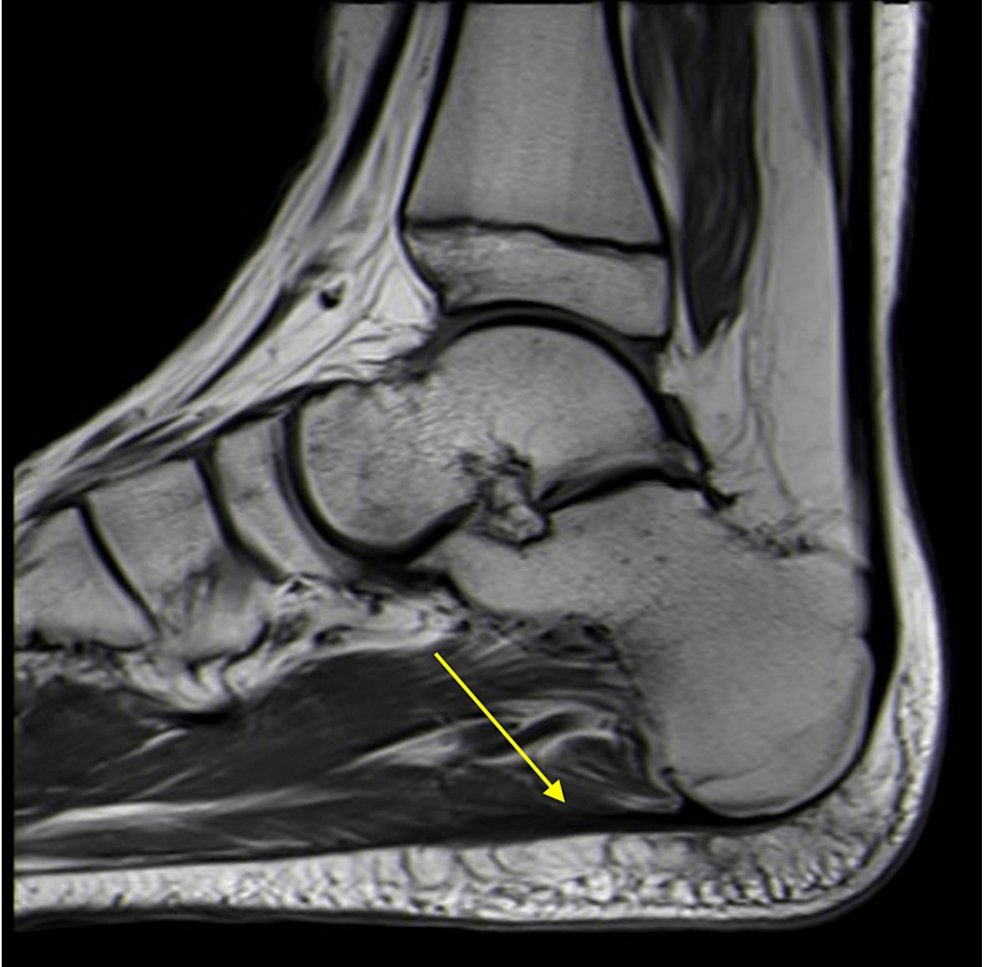
Sagittal T1 weighted MR of the ankle. Imaging demonstrates moderate thickening of the plantar fascia proximally, just distal to the fascial origin, indicative of plantar fasciitis.

Patients with plantar fasciitis will typically present with morning pain and stiffness along with pain during exercise that is relieved by rest. On physical examination, patients will be tender to palpation along the medial aspect of the plantar fascia as it attaches to the anterior calcaneus [19-21]. Radiographs are not useful for diagnosing this condition [20]. MRI can show thickening of the plantar fascia (Figure 5). 

First line of treatment includes conservative management with rest, ice, arch supports, heel pads, non-steroidal anti-inflammatory drugs (NSAIDs), stretching, and strengthening [20,21]. Steroid injections of the plantar fascia in the pediatric population are controversial [20]. Surgical management is reserved for extreme or complex patients who fail conservative management and includes partial plantar fascia release [21].

Calcaneal Stress Fracture

Stress fractures occur due to microscopic injuries sustained when bone is subjected to repeated submaximal stress during training and competition without adequate rest [22]. Adolescents participating in sports are at an increased risk of this injury due to an immature musculoskeletal system and periods of rapid bone growth [23]. Recently, there has been an increase in the number of adolescents participating in year-round athletics. It is well documented that year-round sports can lead to increased incidence of overuse injuries, such as stress fracture [24]. In children and adolescents, stress fractures of the foot and ankle most commonly occur in the metatarsals and the calcaneus followed by the cuboid, talus, and navicular [25]. There should be a high clinical suspicion of this diagnosis in children who have an abrupt increase in activity or who participate in high-impact activities such as running and jumping.

Calcaneal stress fractures typically present with an insidious onset of pain after initiation of an activity. On examination, patients will have tenderness over the body of the calcaneus [26]. Pain elicited by squeezing the calcaneus from both sides simultaneously (positive squeeze test) can usually differentiate this condition from retrocalcaneal bursitis, Achilles tendinitis, plantar nerve entrapment, plantar fasciitis, and heel spur [26]. Initial radiographs are often negative and can be repeated in two to three weeks for greater accuracy. MRI is superior for detecting these fractures early but is not necessary for diagnosis. Radiographs generally become positive within the first month after the onset of pain and show callus formation perpendicular to the trabecular “grain” of the calcaneus, usually located between the calcaneal tuberosity and the posterior facet of the subtalar joint [26]. MRI demonstrates marrow edema and occasionally a fracture line subjacent to the posterior facet of the subtalar joint or the calcaneal tuberosity [26]. 

Most stress fractures are uncomplicated and managed conservatively with activity restriction and gradual return to activity. Healing is typically rapid and return to activity is usually possible in four to six weeks.

Toddler’s Fracture

**Figure 6:**
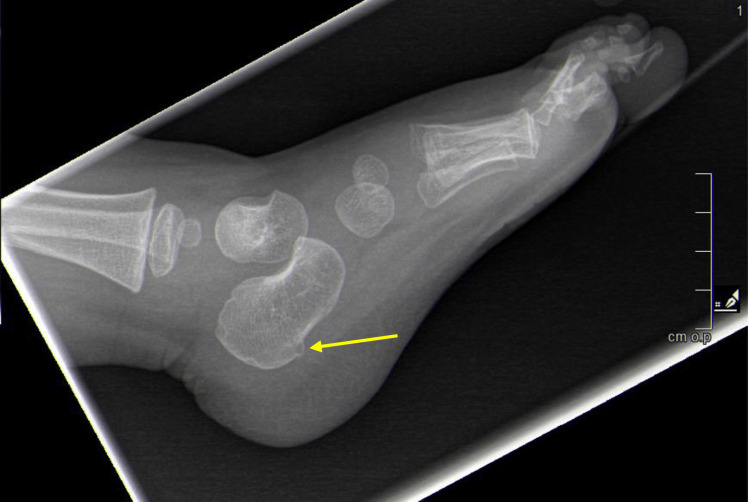
Lateral radiograph of the left foot demonstrating a toddler’s fracture. Patient history notable for limping of three weeks duration. Fracture is located at the plantar aspect of the calcaneus. Note buckle of plantar cortex and subtle periosteal reaction.

The toddler’s fracture was first described by Dunbar in 1964 and originally referred to a non-displaced, spiral fracture of the tibia [27]. The concept of the toddler’s fracture was broadened by John et al. to include fractures of the fibula, metatarsals, talus, cuboid, and calcaneus [28]. Non-displaced fractures of the calcaneal tuberosity are mainly seen in the toddler age group. The typical mechanism of injury is when a toddler jumps from a low height and subsequently refuses to bear weight. Within a few days of the injury, the child will start walking on his/her toes. A clear history of sudden onset after an episode of trauma and tenderness to palpation over the calcaneal tuberosity is suggestive of this injury; however, it is common for a child to present without significant history of injury [29]. Initial radiographs are usually normal but repeat films at two weeks or later will typically show a vertical sclerotic line through the calcaneal tuberosity (Figure 6) [30]. It is important to elicit from the parent an acute onset after trauma because hematogenous osteomyelitis of the calcaneus can occur in this age group. The prognosis of a toddler’s fracture is excellent. Immobilization is not necessary, and symptoms resolve on their own in a matter of weeks.

Osteomyelitis

Osteomyelitis is a common condition observed in the pediatric population. Acute hematogenous osteomyelitis (AHO) is the most common form of osteomyelitis affecting children. AHO commonly presents in healthy children, but certain conditions such as diabetes mellitus, sickle cell disease or trait, hemoglobinopathies, and other immunosuppressive conditions can increase the risk of this ailment. The bacteremia causing AHO is often asymptomatic. AHO usually occurs in the metaphyseal region of long bones but can occur in the foot [31]. The most common pathogen causing AHO is *Staphylococcus aureus* [32-34]. In newborns and children less than two years of age, additional important causes of osteomyelitis include group B *Streptococcus*, *Escherichia coli*, *Klebsiella*, and *Candida albicans*. The etiology of infection is important as puncture wounds through the shoe grow *Pseudomonas* in most cases [35,36]. Osteomyelitis of the calcaneus comprises roughly 4-11% of osteomyelitis cases in children [37-39].

**Figure 7:**
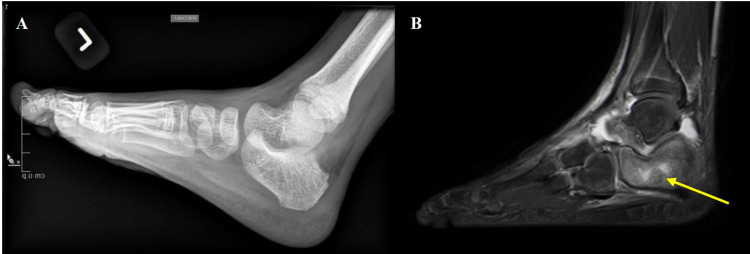
(A) Lateral radiograph of the left foot and ankle. (B) T2 weighted MRI of the foot and ankle. Patient was admitted for limping and heel pain. Plain radiograph is normal. MRI demonstrates extensive edema of the calcaneus, soft tissue inflammation, and fluid in the subtalar joint consistent with osteomyelitis/septic arthritis.

A dictum of pediatric orthopedics is that any child with bone pain and fever should be assumed to have osteomyelitis until proven otherwise. Osteomyelitis typically presents with pain or tenderness at the infected site and fever. Osteomyelitis of the calcaneus is no different and usually presents with heel pain, toe walking, and possibly fever. On examination there may be erythema, swelling, and tenderness to palpation. Radiographs should be obtained but may be normal within the first two weeks (Figure 7a) [40]. CBC, ESR and CRP should be obtained. Inflammation in AHO has been studied, and CRP was determined to be more useful for monitoring recovery and/or response to treatment versus ESR as ESR is slower to normalize [41]. MRI is confirmatory and is useful to determine if there is an abscess requiring surgical drainage or involvement of the subtalar joint (Figure 7b). Blood cultures should be obtained. Aspiration of the affected area is very important for diagnosis, and management and should be performed prior to the initiation of antibiotics. If the child is not markedly ill or septic, it is best to withhold antibiotics until it is determined by orthopedics as to whether a specimen will be obtained for culture. In cases with abscess formation or inadequate response to conservative treatment with antibiotics, surgical irrigation and debridement may be necessary. 

Benign Lesions of the Calcaneus

There are a variety of benign bony lesions that can affect the pediatric population. Lesions affecting the calcaneus are rare with an occurrence as low as 3% of all pediatric bone tumors [42]. Benign pediatric calcaneal lesions can present with pain, mass, and/or swelling. They may also be asymptomatic and found incidentally on imaging. The most common benign lesions affecting the pediatric calcaneus are as follows: osteoid osteoma, simple bone cysts, aneurysmal bone cyst, and osteochondroma.

Osteoid osteoma: Osteoid osteoma (OO) is the most common benign tumor of the foot and ankle accounting for 35% of all foot and ankle tumors. Nine percent of all OO involve the foot and ankle, and up to 75% of cases affect males [42,43]. The calcaneus and talus are the most common sites of OO in the foot [44]. Osteoid osteoma classically presents with severe pain at night relieved by NSAIDs [43]. Radiographs disclose lucency with surrounding sclerosis, which can be misdiagnosed as a simple bone cyst [45]. CT demonstrates a well-defined nidus, which is a round or oval smoothly marginated lytic lesion with central mineralization and is more accurate than MRI for this diagnosis. The mainstay of treatment is surgical excision, which has a high success rate [46]. Medical management with long-term NSAIDs is possible and has been found to be effective; however, surgical excision is the most common treatment modality [47,48]. Recently, less invasive surgical options such as laser and radiofrequency ablation have gained popularity [47]. 

**Figure 8:**
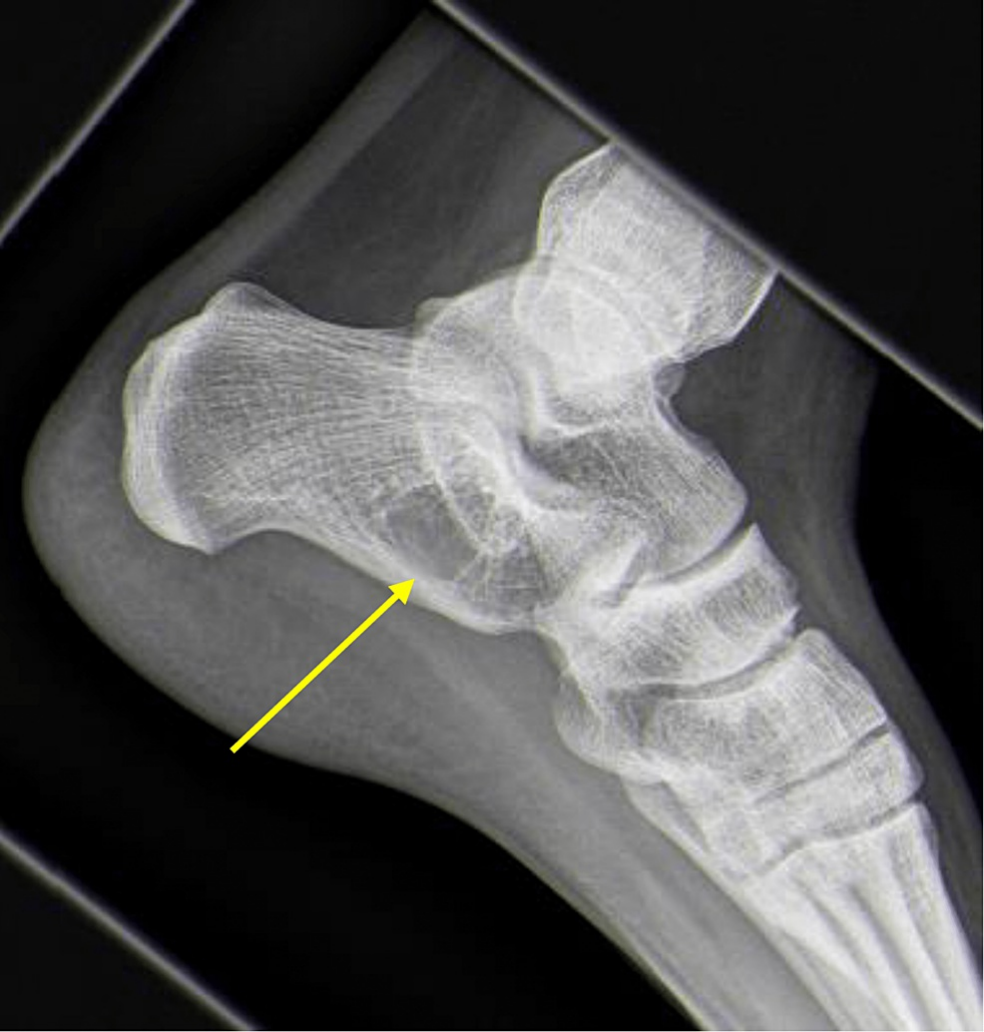
Lateral radiograph of the calcaneus from a 17-year-old patient. Radiograph demonstrates lucent lesion that was incidentally found due to metatarsal fracture from a motor vehicle collision. The lesion was determined to be a simple bone cyst after biopsy with curettage and bone grafting, which were performed due to the risk for pathological fracture.

Simple (unicameral) bone cysts: Simple bone cysts (SBCs) of the calcaneus are often an incidental finding. Studies vary widely in reporting the percent of SBCs occurring in the calcaneus, with some claiming only 4% and others up to 25% [42,49,50]. SBCs occur primarily in children and affect males twice as often as females [51]. Patients are usually asymptomatic but may present with pain [45]. Radiographs disclose lucency and occasionally surrounding sclerosis (Figure 8) [51]. SBCs can be differentiated from aneurysmal bone cysts (ABCs) with characteristic imaging showing a lytic, expansile lesion with a possible irregular or eccentric shape, thin sclerotic borders, and absence of loculation or fluid-fluid levels [42]. The location of the cyst is usually in Ward's triangle of the calcaneus, anterolateral to the posterior facet of the subtalar joint, where physiologic stresses are low (Figures 1, 8). Other pathologies may have a similar plain radiographic appearance, so the differential diagnosis includes aneurysmal bone cyst, intraosseous lipoma, pseudo-cyst, and a cyst within an osseous or chondral tumor [49]. Standard treatment is conservative including NSAIDS, steroid injections, casts, crutches, and extended periods of non-weight bearing [51]. Surgical treatment is not recommended for patients with small, asymptomatic cysts [52]. Larger cysts are typically symptomatic and have a higher predisposition for pathologic fracture [53]. Curettage and bone grafting is a curative surgical option that should be considered for patients with large, symptomatic cysts [53]. 

Aneurysmal bone cyst: Aneurysmal bone cysts (ABCs) are benign, blood-filled, cystic lesions that typically involve the metaphyseal regions of bones. ABCs comprise roughly 1% of bone tumors [54]. They are commonly found in the femur, tibia, spine, humerus, pelvis, and fibula [55-57]. The vast majority of ABCs are seen in patients less than 20 years of age; however, they are rare in children younger than five years old [58]. Most ABCs are primary lesions, but there is a subset of secondary ABCs that are a reactive byproduct of lesions including chondroblastoma, giant cell tumor, and osteoblastoma [59]. It is estimated that only 1.5% of ABCs occur in the calcaneus [55]. Calcaneal ABCs are usually symptomatic. The typical presentation of calcaneal ABC is pain with weight bearing and swelling. Physical examination may show tenderness to palpation, swelling, and a palpable mass. Plain radiographs of the foot are the first line of imaging, which reveals an eccentric, radiolucent, loculated, expansile lesion with thin cortices [60,61]. The commonly eccentric location of ABCs seen on X-ray is a good feature to help distinguish it from unicameral bone cysts, which are typically concentrically located. A lesion with these radiographic characteristics should be evaluated with MRI. Characteristic MRI findings are a cystic lesion with septations and fluid-fluid levels [62]. Biopsy is required for definitive diagnosis. The mainstay of treatment for ABC is curettage and bone grafting. The risk of recurrence is high, making it common practice to use adjuvants such as high-speed burring [63,64]. The risk of malignant transformation is low and is typically found in patients with a history of irradiation [65]. Prognosis is generally good with appropriate surgical treatment [57,66,67]. 

Malignant Lesions of the Calcaneus 

Malignant osseous tumors in the pediatric population are extremely rare, representing roughly 3-6% of all pediatric bone tumors, with malignancies of the foot and ankle comprising a subset of these tumors [68]. Due to their rarity and nonspecific clinical presentation, most cases are diagnosed late. The most common malignancies of the calcaneus in children and adolescents include osteosarcoma and Ewing’s sarcoma. Malignant bone tumors of the calcaneus should be considered in children with persistent heel pain of several weeks duration or children presenting with a soft tissue mass.

## Conclusions

It is important for physicians to be able to recognize the most common causes of heel pain and to know when further workup or referral to a specialist is warranted. The most common cause of heel pain, Sever’s apophysitis, does not typically require special imaging or complicated treatment modalities for diagnosis and resolution. Sever's apophysitis is treated with reassurance, symptom management, and rest. Malignant lesions of the calcaneus are extremely rare. However, it is important to have a high index of suspicion for conditions that do occur with moderate frequency in the foot, such as hematogenous osteomyelitis. 
